# Factors causing erectile dysfunction after bladder neck and nerve sparing robot-assisted laparoscopic radical prostatectomy in men with preoperatively normal erectile function

**DOI:** 10.1007/s11845-026-04317-y

**Published:** 2026-03-31

**Authors:** Selcuk Sarikaya, Turgay Ebiloglu, Mehmet Selcuk Buyantemur, Halil Gurdal Inal, Selahattin Bedir

**Affiliations:** Department of Urology, University of Health Sciences, Gulhane Research and Training Hospital, Emrah Mahallesi, General Tevfik Saglam Caddesi, Etlik, Ankara, Turkey

**Keywords:** Erectile dysfunction, Prostate cancer, Radical prostatectomy

## Abstract

**Introduction:**

Patients with localized prostate cancer(PCa) who undergo radical prostatectomy have an approximately 100% disease-specific survival rate. However, complications such as erectile dysfunction(ED) are common. This study evaluates the factors influencing erectile function following bladder neck and nerve-sparing robot-assisted laparoscopic radical prostatectomy(RARP).

**Materials and methods:**

Between October 2016 and October 2023, 175 localised PCa patients with normal erectile function underwent bladder neck and nerve-sparing RARP. Preoperative and postoperative factors related to erectile function were assessed. Preoperative parameters included age, comorbidities total Prostate-specific Antigen(PSA), free PSA, free/total PSA ratio, digital rectal examination(DRE) findings, prostate Magnetic Resonance Imaging(MRI) findings, PSA density, cognitive fusion prostate biopsy(CFPB) results, and cancer staging. Postoperative parameters included pathology findings, postoperative PSA levels, the necessity for additional treatment, and PSA levels following additional treatment.

**Results:**

Age, perineural invasion and positive surgical margins in postoperative pathology were identified as significant risk factors for postoperative ED(p<0.05). Having DM was also a significant factor for postoperative ED in biochemically recurrence free patients. The age 58 was detected as a cutoff for predicting normal erectile function postoperatively, with 90% sensitivity and 90% specificity. A significant correlation was also observed between positive surgical margins and perineural invasion in postoperative pathology reports(p=0.001), suggesting tumor-related neural invasion.

**Conclusion:**

Patient age and having DM seemed to be the predictable factors for postoperative ED before RARP. Patients younger than 58 years and without DM had a high likelihood of preserving erectile function. The other factors could not be used to assess the postoperative ED.

## Introduction

Prostate cancer(PCa) is a prevalent disease with a favorable prognosis, with patients who undergo radical prostatectomy for localized cancer achieving an approximately 100% disease-specific survival rate [1]. PCa is the most commonly diagnosed cancer in the elderly population and may be the most common non-cutaneous malignancy among men [2]. Retropubic radical prostatectomy remains the gold-standard treatment for locally invasive PCa [3]. 

PCa is diagnosed in a significant proportion of male cancer cases and is managed through various treatment modalities, including watchful waiting, active surveillance, radical prostatectomy surgiries, radiotherapy, hormone therapy, chemotherapy, and other interventions [4]. However, these treatments can lead to complications, such as erectile dysfunction (ED) [4]. Studies have reported a significantly increased risk of ED within 12 months postoperatively [5]. 

Robotic versus open radical prostatectomy operations have been compared in terms of erectile dysfunction and studies revealed slight improvement for erectile function with robot-assisted operations [6]. Today, Robot-Assisted Radical Prostatectomy(RARP) is the most commonly performed technique, but its advantage in preserving erectile function remains inconclusive [3]. Adjuvant radiotherapy has been found to have detrimental effects on erectile function recovery following bilateral nerve-sparing radical prostatectomy [7]. Studies have also reported a significant decrease in penile size following nerve-sparing Radical Prostatectomy (RP) [8]. Treatment with phosphodieasterase type-5 inhibitors(PDE5-Is) after radical prostatectomy is commonly used for preventing erectile dysfunction after radical prostatectomy [2]. Up to 10% of the patients had significant erection with sildenafil after non-nerve sparing radical prostatectomy according to the studies [9]. 

There are several techniques that have been described in order to get best postoperative potency outcomes but there is no consensus or standart technique [10]. Antegrade and retrograde nerve sparing approaches have been reported in the literature [10]. The important point is to preserve much periprostatic fascia(PPF) and atraumatic neurovascular bundle(NVB) dissection [10]. International Index of Erectile Function-5 (IIEF-5) is one of the most common questionnaire for evaluating erectile functions after radical prostatectomy [4]. Studies suggested that nerve-sparing radical prostatectomy should be planned independently of preoperative potency status if oncologically or technically suitable [11]. Also fascia preservation scores, patient’s age, preoperative IIEF scores, Charlson Comorbidity Index scores(CCIS) and the use of surgical clips were found to be predictors for postoperative erectile functions [12]. There are several factors that are affecting erectile functions and cause erectile dysfunction after radical prostatectomy.

In our study, preoperative and postoperative factors affecting erectile function after bladder neck and nerve-sparing robot assisted laparoscopic radical prostatectomy have been evaluated.

## Materials and methods

### Ethics statement

This study was approved by the Ethical Committee of the University of Health Sciences Gulhane Research and Training Hospital (Approval Number: 2023 − 288). The institution’s Review Board of Human Subject Guidelines was followed.

Between October 2016 and October 2023, a total of 500 patients underwent bladder neck and nerve-sparing robotic radical prostatectomy. Among them, 175 patients reported normal erectile function preoperatively and were carefully followed postoperatively. Patients who developed ED after surgery were evaluated to determine potential contributing factors.

### Patient selection and preoperative assessment

Routine annual prostate cancer screening is recommended for the male population. Patients with total Prostate-Specific Antigen (PSA) levels ≥ 2.5 ng/mL were classified as having elevated PSA and underwent a second test for verification at the same diagnostic laboratory.

As part of the screening program, patients with verified elevated PSA and/or abnormal digital rectal examination (DRE) findings who had a life expectancy of more than 10 years were referred for multiparametric prostate MRI (mpMRI) and subsequently for cognitive fusion prostate biopsy (CFPB) if needed.

### Imaging and biopsy procedures

Prostate Multiparametric Magnetic Resonance Imaging (ProstateMpMRI) was performed using a Siemens 3T MRI machine, and findings were evaluated using the PIRADS Version 2.1 scoring system as defined by the European Society of Urogenital Radiology (ESUR) and the American College of Radiology (ACR) in 2023 [13–15]. For central gland lesions, the same version PIRADS scoring system for the transitional zone was used.

Cognitive Fusion Prostate Biopsy (CFPB) was performed by an expert with 10 years of experience using a dual-plane ultrasound device (Logic C5) and a transrectal probe. Standard transrectal ultrasound-guided prostate biopsy (TRUSPB) was conducted with a minimum of 12-core sextant biopsies using an 18-gauge Tru-Cut biopsy needle gun. Additional targeted biopsies were taken based on PIRADS 4 or 5 Prostate MpMRI findings. If the PIRADS score was 1, 2, or 3, and the patients had high likelihood of PCa; targeted biopsies were obtained from the most suspicious areas despite the reduced accuracy of fusion targeting in these cases.

Exclusion criteria for CFPB included patients with a life expectancy of less than 10 years, even if they had elevated PSA and/or abnormal DRE findings. The decision on CFPB or standard TRUSPB for patients having PIRADS 1, 2, or 3 lesions were made according to PSAD and likelihood of PCa according to the EAU guide line.

### Risk classification and staging

Following pathologic evaluation, patients diagnosed with prostate cancer (PCa) were classified according to the D’Amico risk classification [16].

- Patients classified as intermediate- or high-risk underwent staging with Gallium 68(Ga-68) Prostate-Specific Membrane Antigen (PSMA) Positron Emission Tomography (PET) Scan, thoracoabdominopelvic computed tomography (CT) and/or bone scintigraphy.
- Low-risk patients did not undergo routine staging, as per the 2024 European Association of Urology (EAU) guidelines [16].

### Informing the patients about the treatment modalities

Patients with localized or locally advanced PCa were directed to the urooncology counsel. All the treatment modalities were told to the patients including active surveillance for low risk patients; radiotherapy for low, intermediate and high risk patients; RARP for low, intermediate and high risk patients; androgynous deprivation therapy for intermediate and high risk patients; or multimodal regimen combinations of all these modalities. Patients chosen the RARP were operated and included in this study.

### Erectile function assessment

Preoperatively, erectile function was assessed using the International Index of Erectile Function-5 (IIEF-5) questionnaire. The same questionnaire was repeated one year postoperatively to assess erectile function recovery.

- Patients with an IIEF-5 score ≥ 22 were classified as having normal erectile function.
- Patients with an IIEF-5 score ≤ 21 were classified as having erectile dysfunction.

### Postoperative penile rehabilitation

All the patients took daily 5 mg tadalafil and kegel excercises until the postoperative 12 month.

### Data collection and postoperative follow-up

The preoperative factors were recorded:

- Demographics: Age, comorbidity status (smoking, hypertension, diabetes mellitus, additional illnesses).
- Laboratory and Clinical Findings: Total PSA, free PSA, free/total PSA ratio, DRE findings, mpMRI findings, PSA density (MRI-based), CFPB results, and staging outcomes.

The following postoperative factors were recorded:

- Pathology Results: Surgical margin status, perineural invasion, lymphovascular invasion.
- Oncological Follow-up: PSA levels, need for additional treatments (e.g., radiotherapy, androgen deprivation therapy).
- Biochemical Recurrence (BCR): Defined as total PSA ≥ 0.2 ng/mL.

### Statistical analysis

Statistical analysis was performed using SPSS 26.0 for Windows (IBM Corp., Chicago, USA) by an expert biomedical statistician.

- The Shapiro-Wilk, Kolmogorov-Smirnov, Kurtosis, and Skewness tests were applied to assess the normality of variable distributions.
- Descriptive statistics were presented as follows:
  - Nominal variables: Expressed as frequencies (n) and percentages (%).
  - Continuous variables: Since most parameters did not follow a normal distribution, they were expressed as median, mean ± standard deviation (minimum–maximum).
- The Chi-square test was used to compare independent nominal variables.
- The Mann Whitney U Test was used to compare the independent continuous variables without normally distribution.
- Enter Logistic Regression Analysis was performed to identify significant predictors of postoperative erectile dysfunction (ED).
- Receiver Operating Characteristic (ROC) curve analysis was conducted to determine the sensitivity and specificity of patient age as a predictor of postoperative ED.
- A cut-off age was established, corresponding to 90% sensitivity and 90% specificity for maintaining normal erectile function postoperatively.

A p-value < 0.05 was considered statistically significant.

## Results

A total of 175 patients with normal erectile function underwent bladder neck and nerve-sparing robotic radical prostatectomy for localized prostate cancer. The mean age was 68.96 ± 6.59 years (range: 43–75). Among them, 82 (46%) were smokers, 39 (22%) had hypertension, 99 (56%) had diabetes mellitus, 31 (18%) had coronary artery disease, and 10 (6%) had chronic obstructive pulmonary disease. The median PSA value was 7.75 ng/mL, and the mean PSA was 13.07 ± 26.06 ng/mL (range: 2.3–310). The median free PSA was 1.03 ng/mL, and the mean was 1.51 ± 1.97 ng/mL (range: 0.32–20). The median free/total PSA ratio was 11%, and the mean was 13% ± 9% (range: 0–80%) The detailed analysis results for BCR free and BCR positive patient groups are presented in Table 1.

**Table 1:** Demographic factors, laboratory results and other variables in detail

Factors	BCR free	BCR positive	*P*
Age	68.86 ± 6.85 years (range: 43–75)	68.45 ± 7.24 years (range: 55–75)	0.796
Cigarette smoking	Smokers: 65 patients (%49) Non-Smokers: 66 patients (51%)	Smokers: 17 patients (%38) Non-Smokers: 27 patients (62%)	0.382
Hypertension	Having hypertension: 26 patients (19%) Normotension: 105 patients (81%)	Having hypertension: 13 patients (29%) Normotension: 31 patients (71%)	0.319
Diabetes Mellitus	Having diabetes mellitus: 72 patients (55%) Non-diabetics: 59 patients (45%)	Having diabetes mellitus: 27 patients (62%) Non-diabetics: 17 patients (38%)	0.633
Any other chronic illnesses	Yes, having: 56 patients (43%) No, not having: 75 patients (67%)	Yes, having: 29 patients (66%) No, not having: 15 patients (34%)	0.106
Total PSA(ng/ml)	The median total PSA value: 7.36 The mean total PSA value: 10.03 ± 9.46 (range: 2.30–74.74)	The median total PSA value: 10.06 The mean total PSA value: 24.59 ± 61.83 (range: 3.38–310)	0.058
Free PSA(ng/ml)	The median free PSA value: 1.01 The mean free PSA value: 1.23 ± 0.93 (range: 0.32–6.43)	The median free PSA value: 1.09 The mean free PSA value: 2.19 ± 3.97 (range: 0.53–20)	0.332
Free/Total PSA	The median free/total PSA: 12% The mean free/total PSA value: 13% ± 7% (range: 3%–41%)	The median free PSA value: 11% The mean/total free PSA value: 12% ± 6% (range: 6%–12%)	0.422
ProstateMRI PIRADS score	PIRADS 1: 2 patients (2%) PIRADS 2: 29 patients (22%) PIRADS 3: 21 patients (16%) PIRADS 4: 59 patients (45%) PIRADS 5: 20 patients (15%)	PIRADS 1: 1 patients (2%) PIRADS 2: 1 patients (2%) PIRADS 3: 10 patients (22%) PIRADS 4: 12 patients (27%) PIRADS 5: 20 patients (45%)	0.335
PSAD	The median PSAD: 0.19 The mean PSAD: 0.23 ± 0.18 (range: 0.05–1.54)	The median PSAD: 0.24 The mean PSAD: 0.37 ± 0.49 (range: 0.04–2.36)	0.104
Preoperative gleason score	3 + 3: 89 patients (68%) 3 + 4: 25 patients (19%) 4 + 3: 5 patients (4% ) 4 + 4: 7 patients (5% ) 4 + 5: 2 patients (1% ) 5 + 3: 3 patients (3% )	3 + 3: 11 patients (25%) 3 + 4: 5 patients (11%) 4 + 3: 12 patients (27%) 4 + 4: 9 patients (21%) 4 + 5: 5 patients (11%) 5 + 3: 2 patients (5% )	0.126
Preoperative tumor percentage at prostate biopsy	The median tumor percentage: 8% The mean tumor percentage: 19.13% ± 18.3% (range: 5%–80%)	The median tumor percentage: 14% The mean tumor percentage: 21.92% ± 23.51% (range: 6%–90%)	0.069
Lymphovascular invasion at preoperative biopsy	Yes, having: 0 patients (0%) No, not having: 131 patients (100%)	Yes, having: 0 patients (0%) No, not having: 44 patients (100%)	
Perineural invasion at preoperative biopsy	Yes, having: 26 patients (20%) No, not having: 105 patients (80%)	Yes, having: 18 patients (40%) No, not having: 26 patients (60%)	***0.045***
High grade PIN at preoperative biopsy	Yes, having: 9 patients (7%) No, not having: 122 patients (93%)	Yes, having: 7 patients (15%) No, not having: 26 patients (85%)	0.225
Any lesion at preoperative staging techniques	Yes, having: 6 patients (4%) No, not having: 125 patients (96%)	Yes, having: 3 patients (6%) No, not having: 41 patients (94%)	0.678
Postoperative gleason score	3 + 3: 40 patients (30%) 3 + 4: 44 patients (33%) 3 + 5: 2 patients (1%) 4 + 3: 32 patients (24% ) 4 + 4: 8 patients (6% ) 4 + 5: 4 patients (3% ) 5 + 4: 1 patients (< 1%)	3 + 3: 10 patients (22%) 3 + 4: 11 patients (25%) 3 + 5: 2 patients (4%) 4 + 3: 12 patients (27%) 4 + 4: 6 patients (13%) 4 + 5: 3 patients (6%)	0.271
Tumor percentage at postoperative pathology	The median tumor percentage: 8% The mean tumor percentage: 12.32% ± 14.49% (range: 10%–80%)	The median tumor percentage: 15% The mean tumor percentage: 21.86% ± 21.23% (range: 3%–90%)	0.004
***Perineural invasion at postoperative pathology***	Yes, having: 87 patients (67%) No, not having: 44 patients (33%)	Yes, having: 41 patients (95%) No, not having: 3 patients (5%)	***0.05***
Surgical margin positiveness at postoperative pathology	Yes, having: 39 patients (30%) No, not having: 92 patients (70%)	Yes, having: 18 patients (42%) No, not having: 26 patients (58%)	0.243
Lymphovascular invasion at postoperative pathology	Yes, having: 7 patients (6%) No, not having: 124 patients (94%)	Yes, having: 7 patients (16%) No, not having: 37 patients (84%)	0.088
High grade PIN at postoperative pathology	Yes, having: 24 patients (18%) No, not having: 107 patients (82%)	Yes, having: 13 patients (30%) No, not having: 31 patients (70%)	0.183
Seminal vesicula invasion at postoperative pathology	Yes, having: 14 patients (10%) No, not having: 117 patients (90%)	Yes, having: 4 patients (12%) No, not having: 39 patients (88%)	0.496
Bladder neck involvement at postoperative pathology	Yes, having: 2 patients (2%) No, not having: 129 patients (98%)	Yes, having: 0 patients (0%) No, not having: 44 patients (100%)	0.496
Erectile dysfunction at 12th month	Having erectile dysfunction 86 patients (66%) Having normal erectile function: 45 patients (34%)	Having erectile dysfunction 30 patients (68%) Having normal erectile function: 14 patients (32%)	0.867

Digital rectal examination (DRE) findings were abnormal in six patients (3%), with two patients (1%) having a palpable nodule and four patients (2%) exhibiting increased prostate thickness. Prostate MRI revealed PIRADS classifications as follows: three patients (3%) were PIRADS 1, 30 (17%) were PIRADS 2, 31 (18%) were PIRADS 3, 71 (40%) were PIRADS 4, and 40 (22%) were PIRADS 5. The mean prostate volume was 67 ± 42 mL (range: 24–123), and the median PSA density (PSAD) was 0.20, with a mean of 0.29 ± 0.32 (range: 0.04–2.36) The detailed analysis results for BCR free and BCR positive patient groups are presented in Table 1.

Cognitive fusion prostate biopsy (CFPB) revealed Gleason scores of 3 + 3 in 95 patients (55%), 3 + 4 in 30 (17%), 4 + 3 in 19 (11%), 4 + 4 in 18 (10%), 4 + 5 in 8 (4%), and 5 + 3 in 5 (3%). The median tumor percentage in sampled prostatic tissue was 10%, and the mean was 16.7% ± 19.18% (range: 1–90%). Additional findings included perineural invasion in 44 patients (25%), high-grade prostatic intraepithelial neoplasia (PIN) in 16 patients (9%), and intraductal carcinoma in 1 patient (0.5%), with no cases of extraprostatic extension, seminal vesicle invasion, or lymphovascular invasion The detailed analysis results for BCR free and BCR positive patient groups are presented in Table 1.

Based on D’Amico risk classification, 49 patients (28%) with PSA < 10 ng/mL and Gleason 3 + 3 disease were classified as low-risk and did not undergo staging. The remaining 126 patients (72%) were classified as intermediate- or high-risk and underwent staging with Ga68-PSMA-ligand PET/CT scintigraphy, or thoracoabdominopelvic computed tomography (CT) and bone scintigraphy. Ga68-PSMA-ligand PET/CT was performed in 40 patients (22%), revealing pathologic iliac lymph node involvement in six patients (3%) and focal bone metastasis in one patient (0.5%). Thoracoabdominopelvic CT and bone scintigraphy was performed in 86 patients (49%), identifying pathologic iliac lymph node involvement in one patient (0.5%) and focal bone metastasis in one patient (0.5%) The detailed analysis results for BCR free and BCR positive patient groups are presented in Table 1.

Postoperative pathology revealed Gleason scores of 3 + 3 in 50 patients (28%), 3 + 4 in 55 (31%), 4 + 3 in 44 (25%), 4 + 4 in 14 (8%), 3 + 5 in 4 (2%), 4 + 5 in 7(4%), and 5 + 4 in 1(< 1%). Perineural invasion was identified in 128 patients (73%), high-grade PIN in 37 patients (21%), and intraductal carcinoma in 1 patient (0.5%). Positive surgical margins were found in 57 patients (32%), seminal vesicle invasion in 19 (10%), lymphovascular invasion in 14 (8%), and bladder neck involvement in 2 (1%). The median tumor percentage in the prostatectomy specimens was 10%, and the mean was 16.26% ± 17.88% (range: 1–90%) The detailed analysis results for BCR free and BCR positive patient groups are presented in Table 1.

Comparing preoperative CFPB and postoperative pathology, patients with preoperative Gleason 3 + 3 disease had a significant increase to Gleason 4 + 3 (*p* = 0.044), whereas those with preoperative Gleason scores of 3 + 4 or higher did not show significant changes (*p* = 0.571). Overall, the change in total Gleason score was not statistically significant (*p* = 0.117). Other preoperative and postoperative pathological parameters also showed no significant difference (*p* = 0.399).

During follow-up, biochemical recurrence (BCR) was observed in 44 patients (26%) at various time points: 7 at 3 months, 4 at 6 months, 2 at 9 months, 3 at 12 months, 3 at 15 months, and additional cases through 60 months. The remaining 131 patients (74%) remained BCR free. Biochemical recurrence positive patients underwent secondary treatment with radiotherapy and androgen deprivation therapy (ADT), resulting in PSA levels declining to a nadir state.

At 12 months postoperatively, erectile dysfunction (ED) was reported in 116 patients (66%), while 59 patients (34%) maintained normal erectile function (Table 1). Regression analysis of preoperative and postoperative parameters identified that older age, postoperative perineural invasion, and positive surgical margins as significant risk factors for postoperative ED(*p* < 0.05) (Table 2). ROC curve analysis identified age 58 as a cutoff for predicting normal erectile function postoperatively, with 90% sensitivity and 90% specificity. Patients younger than 58 years had a high likelihood of preserving erectile function.

**Table 2:** Possible factors which may cause erectile dysfunction after bladder neck and nerve sparing robotic radical prostatectomy

Factors	P* value	Odds ratio
***Age***	***0.014***	***0.993***
Cigarette smoking	0.380	0.701
Hypertension	0.963	1.083
Diabetes Mellitus	0.538	1.289
Any other chronic illnesses	0.359	1.042
Total PSA	0.183	0.959
Free PSA	0.201	0.706
Free/Total PSA	0.718	2.620
ProstateMRI PIRADS score	0.186	0.741
PSAD	0.058	0.500
Preoperative total gleason score	0.709	0.905
Preoperative tumor percentage at prostate biopsy	0.372	0.989
Perineural invasion at preoperative biopsy	0.601	0.788
High grade PIN at preoperative biopsy	0.370	0.516
Any lesion at preoperative staging techniques	0.741	1.333
Postoperative total gleason score	0.149	0.692
Tumor percentage at postoperative pathology	0.306	0.988
***Perineural invasion at postoperative pathology***	***0.012***	***2.888***
***Surgical margin positiveness at postoperative pathology***	***0.022***	***2.784***
Lymphovascular invasion at postoperative pathology	0.363	1.857
High grade PIN at postoperative pathology	0.849	1.094
Seminal vesicula invasion at postoperative pathology	0.363	1.857
Bladder neck involvement at postoperative pathology	0.610	0.483

*Enter Logistic Regression

**Fig. 1:**
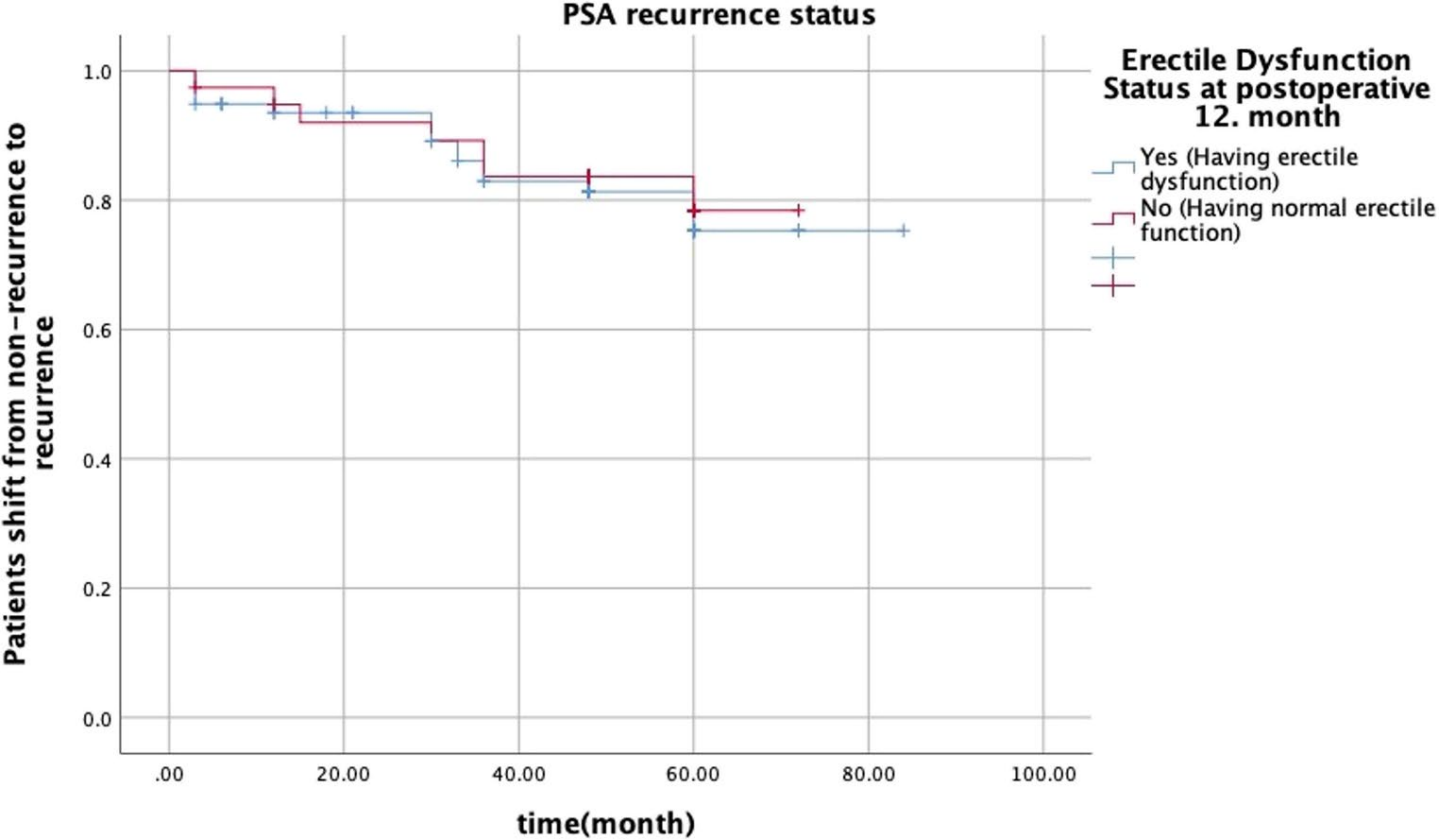
The relation between the PSA recurrence and erectile dysfunction status

Comparing BCR free and positive patients, there was no difference in any parameters (*p* > 0.05) except the perineural invasion rates in preoperative and postoperative pathology (*p* < 0.05) (Table 1). There were 86 (66%) and 30 (68%) patients with ED in BCR free and positive patients, respectively (*p* = 0.867) (Table 1). Additionally, BCR rates were similar between patients with and without postoperative ED at the continuing follow up visits (*p* = 0.742) (Fig. 1). However, regression analysis of preoperative and postoperative parameters in BCR free and positive patients revealed some notable differences: age, having Diabetes Mellitus(DM) and positive surgical margin were significant risk factors for postoperative ED in BCR free patients (*p* < 0.05) (Table 3); but in BCR positive patients, none of these factors were detected to be significant (*p* > 0.05) (Table 4). In BCR free patients, ROC curve analysis identified age 59 as a cutoff for predicting normal erectile function postoperatively with 90% sensitivity and 90% specificity; but in BCR positive patients, age 56 was detected as a cutoff. Patients younger than mentioned ages above had a high likelihood of preserving erectile function in BCF free and positive patients, respectively.

**Table 3:** Possible factors which may cause erectile dysfunction after bladder neck and nerve sparing robotic radical prostatectomy in BCR free patients

Factors	P* value	Odds ratio
***Age***	***0.019***	***0.922***
Cigarette smoking	0.279	0.620
Hypertension	0.940	5.333
***Diabetes Mellitus***	***0.019***	***0.339***
Any other chronic illnesses	0.169	0.547
Total PSA	0.787	0.997
Free PSA	0.700	0.880
Free/Total PSA	0.707	0.293
ProstateMRI PIRADS score	0.433	0.820
PSAD	0.453	0.325
Preoperative total gleason score	0.445	0.748
Preoperative tumor percentage at prostate biopsy	0.667	1.000
Perineural invasion at preoperative biopsy	0.752	0.833
High grade PIN at preoperative biopsy	0.218	8.640
Any lesion at preoperative staging techniques	0.653	1.957
Postoperative total gleason score	0.460	0.780
Tumor percentage at postoperative pathology	0.304	0.981
Perineural invasion at postoperative pathology	0.752	0.833
***Surgical margin positiveness at postoperative pathology***	***0.041***	***3.105***
Lymphovascular invasion at postoperative pathology	0.379	2.672
High grade PIN at postoperative pathology	0.472	0.674
Seminal vesicula invasion at postoperative pathology	0.767	1.293
Bladder neck involvement at postoperative pathology	0.816	1.985

*Enter Logistic Regression

**Table 4:** Possible factors which may cause erectile dysfunction after bladder neck and nerve sparing robotic radical prostatectomy in BCR positive patients

Factors	P* value	Odds ratio
Age	0.089	0.689
Cigarette smoking	0.476	0.450
Hypertension	0.094	1.083
Diabetes Mellitus	0.114	0.205
Any other chronic illnesses	0.605	1.667
Total PSA	0.419	0.914
Free PSA	0.487	0.612
Free/Total PSA	0.225	1.873
ProstateMRI PIRADS score	0.865	1.189
PSAD	0.069	0.300
Preoperative total gleason score	0.193	0.320
Preoperative tumor percentage at prostate biopsy	0.561	0.985
Perineural invasion at preoperative biopsy	0.388	3.000
High grade PIN at preoperative biopsy	0.997	1.000
Any lesion at preoperative staging techniques	0.832	0.957
Postoperative total gleason score	0.732	0.827
Tumor percentage at postoperative pathology	0.647	1.010
Perineural invasion at postoperative pathology	0.388	3.000
Surgical margin positiveness at postoperative pathology	0.426	2.187
Lymphovascular invasion at postoperative pathology	0.747	1.500
High grade PIN at postoperative pathology	0.364	3.000
Seminal vesicula invasion at postoperative pathology	0.957	0.923
Bladder neck involvement at postoperative pathology	0.096	0.467

* Enter Logistic Regression

No significant difference in ED risk was observed based on the location of positive surgical margins (*p* = 0.837). Age was not predictive of surgical margin positivity (*p* = 0.694).

## Discussion

Postoperative erectile dysfunction is a significant concern for patients undergoing radical prostatectomy, as it greatly impacts their quality of life. Patients are highly apprehensive about the possibility of experiencing ED following surgery [17]. Previous studies have suggested that higher Gleason scores may negatively affect preoperative erectile function [17]. While penile rehabilitation is recommended for all patients, younger individuals tend to achieve better outcomes and faster recovery [17]. In our study, patient age was found to be a statistically significant factor influencing postoperative ED.

Preoperative ED has been associated with lower overall survival and an increased risk of mortality from non-prostate cancer causes after radical prostatectomy [18]. Men with ED should be assessed for cardiovascular risk factors, as preventive strategies may help reduce the likelihood of cardiovascular events [18]. However, in our study, additional comorbidities such as hypertension, diabetes mellitus, and other chronic diseases were not statistically significant predictors of postoperative ED. Karaca et al. reported age, Charlson Comorbidity Index, preoperative IIEF, obturator internus thickness, intraprostatic urethral length, surgical technique, and the degree of nerve preservation were significant to forecast the postoperative ED [19]. However, they did not mention the postoperative patology results in their study. Brajtport et al. reported the age as a significant factor for posoperative ED, and yielded 60 years as a cut off age for having ED after RP [20]. Alemozaffar et al. reported younger age, fewer comorbid conditions, lower prostate-specific antigen (PSA) level, lower cancer severity/risk category, pre-treatment potency, better (higher) pre-treatment EPIC-26 sexual HRQOL score, better (lower) pre-treatment American Urological Association Symptom Index, and plan for nerve-sparing surgical technique were associated with decreased postoperative ED for RP [21]. Bhat et al. reported that the age and nerve sparing status as significant determitants for postoperative sexual status after RARP and the age of 55 years was reported as a cut off [22]. The studies above had similar results but had heterogeneous patient groups. In our study, we especially intended to homogenize patients that have preoperative normal sexual functions.

In our study, several preoperative factors were analyzed for their potential association with postoperative ED including total PSA levels, free PSA levels, free/total PSA ratio, Prostate MpMRI PIRADS scores, PSA density, preoperative Gleason score, preoperative tumor percentage in prostate biopsy, perineural invasion, and high-grade prostatic intraepithelial neoplasia (PIN). Among these, only age and having DM were found to be a statistically significant predictor of postoperative ED in BCR free patients. We also detected that the age 58 as a cut off ED after RARP in general. Patients younger than 58 years had a high likelihood of preserving erectile function. In detail, the cutoff ages were identified as 59 years for biochemically recurrence–free patients and 56 years for biochemically recurrence–positive patients. This difference suggests the detrimental effects of additional treatment modalities, as patients were adversely affected by these treatments despite being younger. In addition, the presence of diabetes mellitus (DM) was a significant factor for postoperative erectile dysfunction (ED) in biochemically recurrence–free patients. Diabetes mellitus is a well-known risk factor for ED and also negatively affects erectile function after robot-assisted radical prostatectomy (RARP).

Additionally, postoperative pathological factors were also evaluated in detail. Postoperative total gleason score, lymphovascular invasion, high-grade PIN, seminal vesicle invasion, bladder neck involvement and biochemical recurrence were not significantly associated with postoperative ED. Positive surgical margins in postoperative pathology were identified as the only significant risk factor for erectile dysfunction both in the overall cohort and in biochemically recurrence–free patients. In biochemically recurrence–positive patients, none of the evaluated factors were found to be significantly associated with ED. Although the rates of ED appeared similar between BCR free and BCR positive patients, these findings clearly demonstrate the detrimental effects of additional treatment modalities on patients.

Various treatment strategies have been proposed for managing postoperative ED and facilitating penile rehabilitation. Phosphodiesterase type 5 inhibitors (PDE5-Is) remain the primary treatment option for ED following bilateral nerve-sparing radical prostatectomy [1]. Studies have demonstrated that PDE5-Is offer better outcomes compared to placebo, although their effects often diminish after discontinuation [3]. Subgroup analyses have indicated that daily use of PDE5-Is results in fewer side effects compared to on-demand use [23]. Specifically, daily udenafil (75 mg) has shown efficacy in improving erectile function following bilateral nerve-sparing robot-assisted laparoscopic prostatectomy (BNS-RALP) [24]. Additionally, avanafil (200 mg) taken on demand has been identified as the most effective PDE5-I for treating ED after nerve-sparing radical prostatectomy (NSRP) [2]. 

However patients with venogenic ED were shown to have limited response when comparing to the patients with arteriogenic ED, Tadalafil 5 mg daily intake was well-tolerated and signifigantly improved erectile functions [25]. Studies showed the efficacy of regular regimen of PDE5-Is for short term treatments and both regular and on-demand regimens for long term treatments [26]. Also Vacuum erection device, intracavernous injection and medicated urethral system for erections have also been pointed as promising treatment options [3]. Stem cell therapy is a promising and effective option for various diseases [27]. Autologous Adipose-Derived Regenerative Cells(ADRC) have been shown as safe and effective treatment options for erectile dysfunction after radical prostatectomy [27]. Low-intensity shockwave therapy (Li-ESWT) was shown as a safe treatment option that improves erectile functions and the therapy increased IIEF-5 and EHS scores after radical prostatectomy [28]. 

Pelvic floor muscle training has been shown to improve quality of life after RP, with positive effects on continence and penile rehabilitation [29]. Preoperative physiotherapeutic interventions utilizing biofeedback protocols have also proven effective in reducing the incidence of postoperative ED [30]. 

In our reserch, we used 5 mg daily tadalafil together with kegel excercises for all patients. Due to the over patient load of our urodynamic laboratory, we had diffuculty for adressing all patients to the biofeedback therapy for pelvis floor muscle training, but we used the kegel excercises.

*Limitations;*

- The sample size may be thought as a limitation.

## Conclusion

Our study reveals important outcomes related with RARP operations. Patient age and having DM seemed to be the predictable factors for postoperative ED before RARP. Patients younger than 58 years and without DM had a high likelihood of preserving erectile function. The other factors could not be used to predict the postoperative ED. Additional treatments for BCR seemed to worsen the ED in an insignificant manner. Studies including large patient populations are needed to support these findings.

## Data Availability

The data of infertile male patients has been generated and analysed with using the hospital records and patient statements. Additional data are available from the corresponding author on reasonable request.
